# Cholera Infantum

**Published:** 1879-08

**Authors:** Preston B. Scott


### SELECTIONS.

---

### CHOLERA INFANTUM.

REMARKS, WITH NOTES OF CASES BY PRESTON B. SCOTT, M. D.

AT the last meeting I could only make a brief reference to the treatment of cholera infantum. I regret a longer time is not allowed me, in this season of unusual opportunities, for more extended observations of the course and management of this disorder. Short as it is, enough has been seen to verify the statements made, enough in a week to strengthen confidence, and the conviction that it is, though an annual Herod among our innocents, yet one of the most preventable and curable of our acute infantile disorders. There is certainly too much general apathy to this annual decimation of the nurslings of the nation. There is individual submissiveness, rather to be deplored, in the common acceptation that this affection is of the inevitably destructive form, and that "after all the blessed child is better off." Cholera infantum is, theorize as you will as to pathology, really a disease of season. Like unto sunstroke in adults, it must be guarded against for a brief time only. In some cases the attacks will surprise a most vigilant sentinel. Generally there is admonitory diarrhœa. Because milk is the proper food of all childhood, there is no reason why this staff of infant life may not be laid aside for a few days of digestive rest. One gushing serous stool will exhaust more than a dozen bottles of milk will restore. Whence comes this gushing flow of the life blood of the child?

The milk which before has been finely curdled, now lies there an oppressive mass, or passes in putrefactive ferment into the bowels. There is the undue heat belonging to this process. The lacteal absorbents receive no proper chyle for the lymph channels. The laws of Dutrochet have changed their balance, and in exosmosis there is the rapid serous waste. The liver and all the intestinal glands are in a state of temporary derangement.

In most of the cases, if the patient can gain a few hours of rest you will command the situation, reorganize the scattered forces, and regulate the secretions. What is his work? In many cases he has the history and phenomena of sunstroke before him—cold to the head, cold and grateful drinks to the stomach; a tepid or cool pack to the body, impressing the nervous system through the sentient nerves of these surfaces. He must at once oppose instinct by separating mother and child for a time. The solution of albumen and gelatine on crushed ice will answer the cravings of thirst and hunger. They do not ferment, they favor the prompt resumption of endosmosis. While in the cases I have noted calomel was the chief medicinal reliance, whiskey, camphor and ether fulfill most of the indications of extreme cases. Opium is too often found to have been placed in the way. The handy laudanum or paregoric vial has already been drawn upon. Here I will only impress two points of familiar notice. The very medicine which is the least indicated and the most capable of harm, is the first resorted to. The food which the disturbing forces have converted into conditions most favorable for their action is stubbornly persisted in, and as obstinately returned also. Opium, hurtful to the child inversely with age, is used by popular hand, with a confidence which, with the profession would be counted sheer recklessness. Milk, the very food on which this ferment is feeding, is given under the eye which sees each portion of it vomited and purged, as often as it is taken. It is a familiar saying, in the story of the case, that as often as it takes the breast or the bottle, it vomits. It is equally familiar to the doctor that it does *not* when it quits the bottle and the breast, and uses only crushed ice and egg water. In the most favorable recovery the return of digestive power of the child is not promptly equal to the ordinary diet.

The common error is in the too early return to this. It is a good rule to suspend altogether cow's milk, and to restrain nursing to longer intervals and smaller quantities, so long as the disorder lasts, and the green curdled masses appear in the stools. It is better not to resume the milk while there is fever, thirst, high-colored urine, sour, green, thin actions. There is nourishment, safety, and generally acceptability in the range of barley water, whey, albumen, gelatine, *panado*, and delicate animal broths. The following cases constitute what I have seen of this affection this season. I have only one other of severe bowel disease, and this, a case of colo-enteritis, is now running an unfavorable course.

Case I. Age four months; milk fed. In good health, digestion and bowels natural until the morning of June 13. Seized 11 A. M. by vomiting and profuse rice-water purging. At 1 o'clock P. M., waste and exhaustion had been rapid, eyes and fontanelles sunken; extremities cold; body hot; thirst and restlessness intense; pulse 150; temperature 104°. Gave a teaspoonful of brandy in iced egg water, and at once wrapped the child in the cradle sheet wet with tepid water and applied ice-cold cloths to the head. In ten minutes the child was quietly sleeping in the pack. Twenty minutes afterwards the temperature was taken and a reduction of two degrees had taken place. The wet sheet was removed and an eighth of a grain of calomel was placed on the tongue, and iced egg water given. Two hours of quiet sleep then followed. Calomel was again given and repeated at bed-time. There was no return of vomiting and one loose action from the bowels occurred in the night. The next morning the temperature was normal, the thirst gone and the child revived. One action, dark and of more natural odor, took place in the afternoon. Egg and rice water only were allowed. On the second day cream was given in rice water. Since then the child has fed on racahout, and has had no trouble with the bowels, and has gradually regained its flesh and strength.

Case II. June 15th; six months old; fed cow's milk; for several days has had loose bowels and occasional vomiting of milk. 5 P. M., had been vomiting and purging all day; pulse 150; temperature 103½ Fahr.; intense thirst; exhausted and restless. At once placed it in tepid pack and applied cold to the head. Gave egg-water on crushed ice. Up to my visit it was allowed the bottle of milk, and at every taking it vomited and purged. In the pack the child became quiet, and rested for half an hour. The temperature came down three-fourths of a degree at 10 P. M. I saw it again. It had taken two doses of one-tenth of a grain of calomel; had not vomited or purged, and had not been so thirsty. It was pretty quiet until 8 P. M. when, growing restless, it was again placed in the wet sheet, and allowed to remain in it half an hour. I found it in a tranquil sleep; pulse 110, and temperature 100½ Fahr. On the morning of the 16th I found it had passed a quiet night, had not vomited, had had one loose, dark stool. Two doses of calomel had been given, egg-water and gelatine were ordered. I saw it on the 17th and learned it had rested well; had a dark pasty stool. I then placed it on Gerber's food and discharged the case.

Case III. June 16; thirteen months old; had six incisors; is nursed and fed. This child was seized with vomiting and purging; stools thin, green, sour smelling, and profuse. Rapid exhaustion coming on, it was not allowed to nurse, and ordered to have egg-water and one-twelfth of a grain of calomel every hour. On the second day dry cracker and panado were allowed. Carbolized camphor water was used to correct tendency to fermentation. On the third day the child was permitted to nurse morning and night. On the fourth day it was dismissed.

Case IV. Nine months old; nursing. Had been purging and vomiting for four hours before my visit at 9 P. M. The child nursed and vomited by rapid turns, followed at short intervals by rice-water stools. The eyes were sunken; extremities cold; thirst insatiable; temperature 102°. Ordered to be taken from the breast and to have iced egg-water *ad lib.* Calomel in 1-16 grain doses every half hour. The vomiting ceased in less than an hour. The intervals between stools grew longer, and the actions, though thin, smaller in quantity. The egg-water was exclusive diet and drink for two days. On the second day the actions were dark and pasty. On the third day the child was allowed to nurse, and is now quite well.

Case V. Age twelve months; at the breast. I saw it at 4 P. M., June 14. Nursing and vomiting and purging from 9 A. M. to the time of my visit. Intense thirst and restlessness; extreme exhaustion; profuse rice-water actions; pulse 150; temperature 99°. I at once had the child taken from the breast, and ordered 1-12 of a grain of calomel every half hour, and egg-water *ad lib.* At 11 P. M. had not vomited; two small, thin actions; less thirst and some sleep. 15th, had passed a good night; was still thirsty, and clamorous for the breast. During the day took carbolized camphor and cinnamon-water and egg-water. In the afternoon had two small, dark, thin actions. 16th, had a natural alvine action. Recovery was now completed, and the case dismissed.

Case VI. Two years old. At the breast. June 18th and 19th. A parallel case in onset, course, and treatment and result, with case 5.

Case VII. June 12th. Twelve months old; fed on cow's milk by day, and condensed milk at night. Vomiting and purging every few minutes. Treated with egg-water and one-twelfth grain dose of calomel every half hour. Twice in the convalescence of this child it was allowed to take condensed milk, with recurrence once of vomiting and diarrhœa, and once of diarrhœa alone, which was relieved by carbolized camphor and cinnamon-water.

Case VIII. June 13th. Five months old; fed on cow's milk; is hearty and vigorous; had sour milk on the 10th and 11th; early in the morning vomiting and purging came on. Thin, curdled milk actions five or six times. 11th and 12th, had occasional vomiting of milk. 13th, vomiting, rice-water purging. Seen 4 P. M. in state of great exhaustion. Treated by small doses of calomel, and the egg and rice-water, with favorable progress until the third day, when it was fed on condensed milk against my instructions. Actions previously small and at long intervals, became frequent, large and sour smelling. Relieved by carbolized camphor mixture, and put on Gerber's food on the sixth day, and has since done well.

Case IX. June 16th. Two years old; in good health, and on general food. Took milk for supper, and ate some berries. Severe vomiting and purging at midnight, and exhausted by three immense watery, offensive actions. Treated with calomel in one-eighth grain doses, iced egg-water and a mixture of carbolic acid, comp. spirits ether and camphor-water. The subsequent diet was rice-water drink, broth of chicken and dry crackers.

Case X. (Mother had lost three preceding children with cholera infantum under ten months. One nursed and the other two were fed with cow's milk to hour of death.) Ten month's old; fed on cow's milk; for two days had loose, curdy, offensive actions. Awoke June 22d, vomiting and serous purging; gave one-tenth grain doses calomel every half-hour, cinnamon, camphor, and carbolic acid water. Dismissed on third day with returning healthy stools. Kept on egg-water for two days, and on third day allowed crackers and chicken soup.—*The Medical Home, Louisville, July, 1879.*

